# Abstract of Proceedings of the Buffalo Medical Association

**Published:** 1869-12

**Authors:** Frank W. Abbott

**Affiliations:** Secretary, pro tem.; Buffalo


ART II —*Abstract of Proceedings of the Buffalo Medical Association:*

BUFFALO, Tuesday Evening, Dec. 7th, 1869.

The President being absent, the meeting was called to order by the Vice-President. Dr. ABBOTT was appointed as Secretary, *pro tem.*

Members present: Drs. STRONG, SAMO, WHITE, CRONYN, BURGER, TROWBRIDGE, PHELPS, WETMORE, DIEHL, ABBOTT.

Dr. Diehl, the Treasurer, reported that the treasury was overdrawn, and that several bills had been presented which required immediate settlement.

Dr. Abbott said that, at the next meeting of the Association, he would read a paper on the Accommodation and Refraction of the Eye, and illustrate the anomalies of refraction—myopia, hypermetropia and astigmatism—by means of a camera.

Dr. John J. Burke was proposed as a member of the Association by Dr. Diehl.

Dr. Strong reported a case of rupture of the uterus, resulting in recovery, which occurred under his observation in Washington. The patient, a primipara, did not have a very severe labor; but before the head reached the superior straight, the pains suddenly ceased, the head receded, and, upon examination, it was found that the child had escaped through a rent in the uterus into the peritoneal sac. The feet were seized and the body delivered, but it was necessary to apply the forceps to deliver the head. The patient recovered, and a careful examination, made five or six weeks afterwards, revealed no trace of the injury.

The case was reported on account of the extreme rarity of recovery after so grave an accident. The records of about 44,000 cases of labor, show sixty-five cases of rupture of the uterus, and only six or seven recoveries.

Dr. White said, that the woman who had a rupture of the uterus whom he saw, in consultation with Dr. Hawley some years since, and whose case was fully reported at the time in the *Buffalo Medical and Surgical Journal*, has since had a natural labor.

He thought that this accident was not so necessarily fatal as was generally supposed, and that the record of succeeding years would show many more recoveries than those of previous years. The essential thing in the treatment, was to remove the child at once, even though it should be necessary to resort to the Cæsarean section. A wound in the peritoneum is by no means certainly fatal.

He saw a case in Bartholomew's Hospital where a child had escaped into the cavity of the abdomen, through a ruptured uterus, two years and a half before. At the time of his examination there was a hard tumor in Douglass' *cul de sac*, which he recognized as a fœtal head. He assisted Dr. Greenhalgh, who cut down upon it through the posterior wall of the vagina, applied the forceps, and extracted the withered mummified body of a child. The patient made a good recovery.

Dr. CRONYN said, that the gravity of this accident depends on where it occurs, where and how it is relieved. It is most dangerous where the rupture occurs at the fundus or posteriorly. There is not much hemorrhage unless the rupture occurs through the attachment of the placenta. He thought that the os uteri was ruptured more frequently than was generally imagined. He had a patient who, in a previous labor, had suffered a laceration of the cervix to the extent of an inch and a half. As a consequence, she had several abortions, but he finally succeeded in closing the rent. She afterwards passed through a labor in safety, under his attendance; but, becoming pregnant again, she employed a midwife and died in labor.

Dr. WHITE made a motion that Dr. BURGER be requested to exhibit his Laryngoscope to the Association at some of its sessions.

Dr. BURGER said it would be impossible to move it and exhibit it satisfactorily in the Society's rooms, but invited the members of the Association to visit his office, where he would be pleased to show it to them.

Meeting adjourned.

FRANK W. ABBOTT, M. D.,
Secretary, *pro tem.*

